# Supplementary material for: Nerve Growth Factor Induces Proliferation and Aggressiveness in Prostate Cancer Cells
Source: Cancers (Basel). 2019 Jun 6;11(6):784. doi: 10.3390/cancers11060784 (PMC6627659; doi:10.3390/cancers11060784)
Supplement: Supplementary file 1 [file cancers-11-00784-s001.pdf]

# Supplementary materials

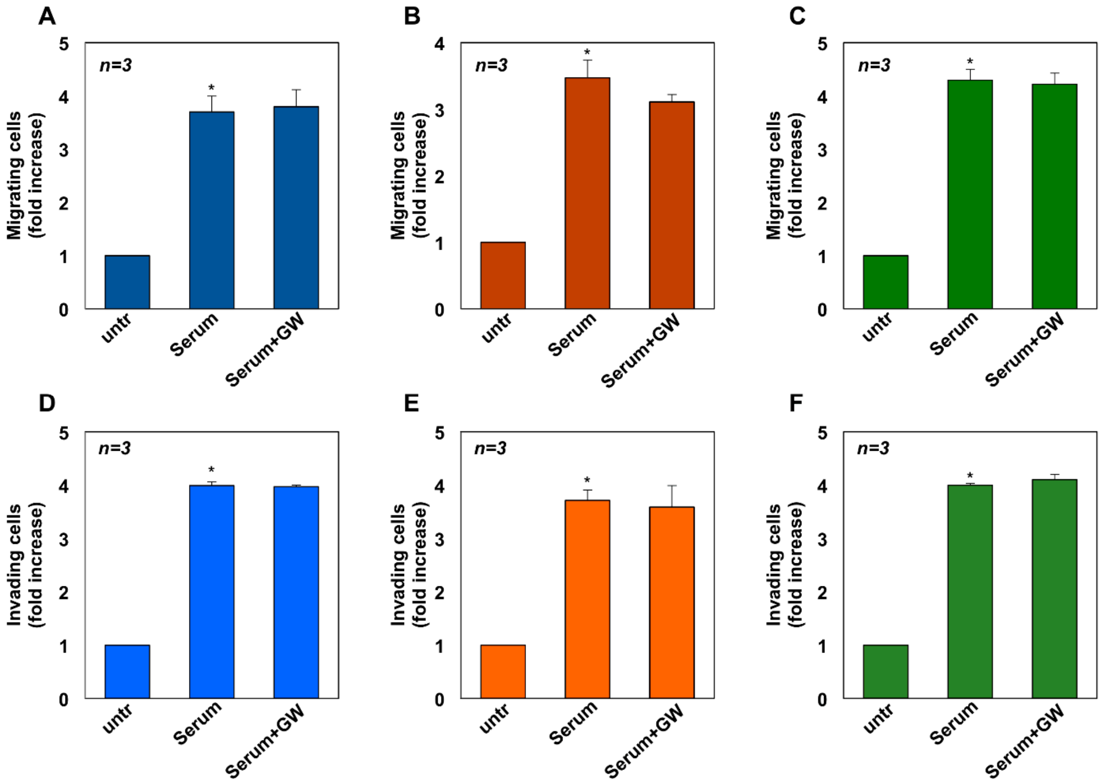


**Figure S1.** Shows migration and invasion assays in CRPC cell stimulated with serum, in the absence or presence of GW441756 (GW). Quiescent C4-2B (**A**, **D**), DU145 (**B**, **E**) and PC3 (**C**, **F**) cells were used for migration (**A**–**C**) or invasion (**D**–**F**) assays in Boyden's chambers pre-coated with collagen or Matrigel, respectively. The indicated compounds were added to the upper and the lower chambers. Serum was used at 20% (v/v) and GW441756 (GW) was used at 1µM. Cytosine arabinoside (at 50 µM) was included in cell medium. After 7 h (**A**–**C**) or 24 h (**D**–**F**), cells were counted as reported in Methods. Results from three different experiments were collected and expressed as fold increase. Means and SEMs are shown. n represents the number of experiments. *: p <0,05 for the indicated experimental points versus the corresponding untreated control.

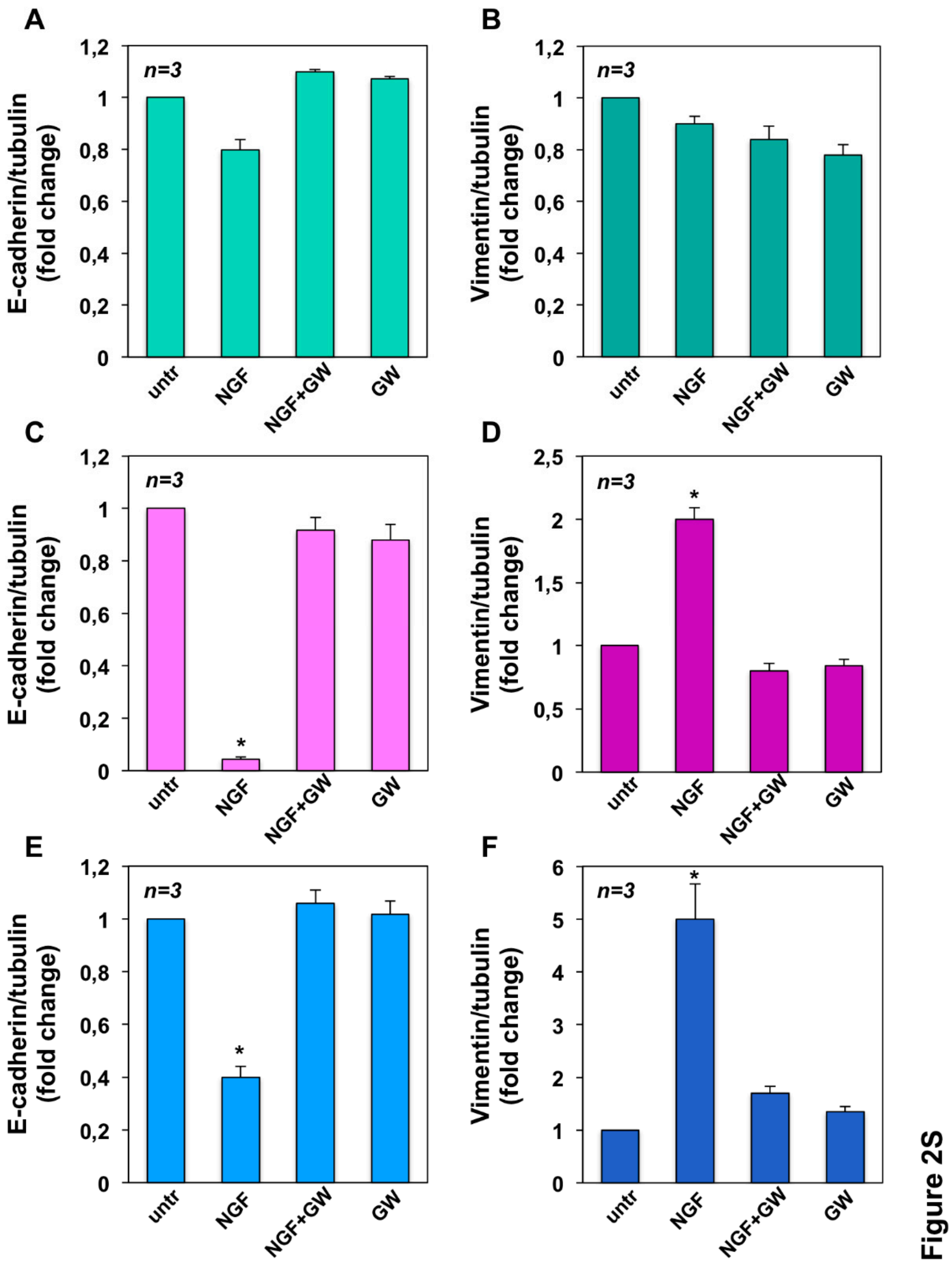


**Figure S2.** Shows the densitometry analysis of EMT markers expression in CRPC cells. Quiescent C4–2B, DU145, and PC3 cells were left untreated or treated for 72h with the indicated compounds. NGF was used at 100ng/mL and GW441756 (GW) at 1 μM. Lysate proteins were analyzed by Western blot, using the antibodies against E-cadherin or vimentin. The blots from three different experiments were done and expression levels of E-cadherin and vimentin were analyzed by densitometry, using NIH Image J Software. For each experiment, the ratio E-cadherin/tubulin in C4-2B (**A**), DU145 (**C**) and PC3 (**E**) cells and the ratio vimentin/tubulin in C4-2B (**B**), DU145 (**D**) and PC3 (**F**) cells was evaluated. Results were expressed as fold change. Means and SEMs are shown, n represents the number of the experiments. *: $p < 0{,}05$ for the indicated experimental points versus the corresponding untreated control.

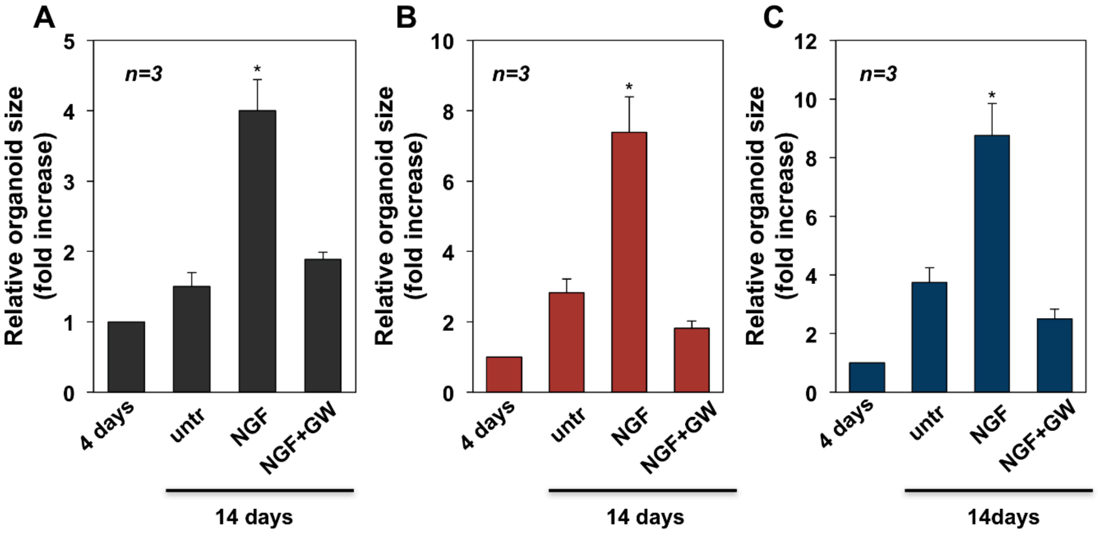


**Figure S3.** Shows the analysis of organoid size from CRPC cells challenged with NGF. Miniaturized 3D C4-2B (**A**), DU145 (**B**) and PC3 (**C**) cultures in ECM were done. The area of organoids was calculated using Leica suite software. For each cell line, 3 different experiments, each in triplicate, were done and results were expressed as fold increase over the basal level analyzed after 4 days. Means and SEMs are shown, n represents the number of the experiments. *: p < 0,05 for the indicated experimental points versus the untreated controls.

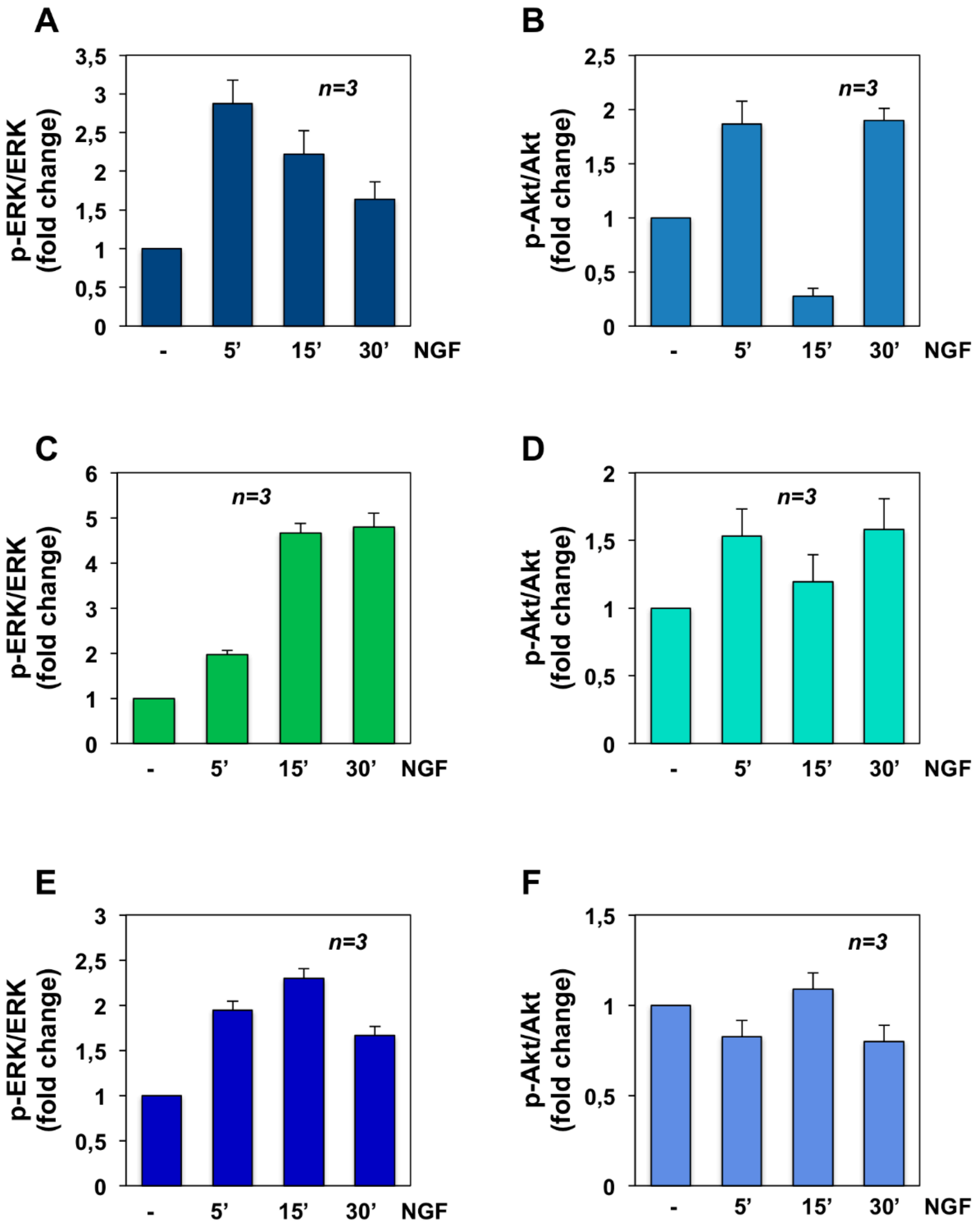


**Figure S4.** Shows the densitometry analysis of ERK and Akt activation in CRPC cells. Quiescent C4-2B, DU145 and PC3 cells were used. Cells were left un-stimulated or stimulated with 100 ng/mL NGF. Lysate proteins were analyzed by Western blot, using the anti p-ERK (P-Tyr 204 ERK 1 and the corresponding phosphorylated ERK 2) or anti p-Akt (P-Ser 473 Akt) antibodies. Filters were re-probed using anti ERK or anti Akt antibodies, as a loading control. Densitometry analysis from 3 different experiments was done using NIH Image J Software. For each experiment, the ratio p-ERK/ERK in C4-2B (**A**), DU145 (**C**) and PC3 (**E**) cells and the ratio p-Akt/Akt in C4-2B (**B**), DU145 (**D**) and PC3 (**F**) cells were evaluated and results expressed as fold change. Means and SEMs are shown, n represents the number of the experiments.
